# [Corrigendum] Puerarin alleviates the ototoxicity of gentamicin by inhibiting the mitochondria-dependent apoptosis pathway

**DOI:** 10.3892/mmr.2026.13849

**Published:** 2026-03-19

**Authors:** Ping Niu, Yuxuan Sun, Shiyi Wang, Guang Li, Xiaomin Tang, Jiaqiang Sun, Chunchen Pan, Jingwu Sun

Mol Med Rep 24: 851, 2021; DOI: 10.3892/mmr.2021.12491

Following the publication of the above article, an interested reader drew to the authors’ attention that, concerning the TUNEL assay data shown in Fig. 5A on p. 6, the images shown for the H_2_O_2_ and PU+H_2_O_2_ groups were remarkably similar, suggesting that these data had been derived from the same original source where the results of differently performed experiments were intended to have been portrayed.

After having re-examined their original data, the authors have realized that an unintentional error was made during the assembly of the images in Fig. 5A; specifically, the representative images for the H_2_O_2_ and PU+H_2_O_2_ groups were inadvertently duplicated. A revised version of Fig. 5, now showing the correct data for the H_2_O_2_ group in Fig. 5A, is shown on the next page. Note that the error made in assembling the data in Fig. 5 did not significantly affect either the results or the conclusions reported in this paper. All the authors agree with the publication of this Corrigendum, and are grateful to the Editor of *Molecular Medicine Reports* for allowing them the opportunity to publish this; moreover, they apologize to the readership for any inconvenience caused.

## Figures and Tables

**Figure 5.**
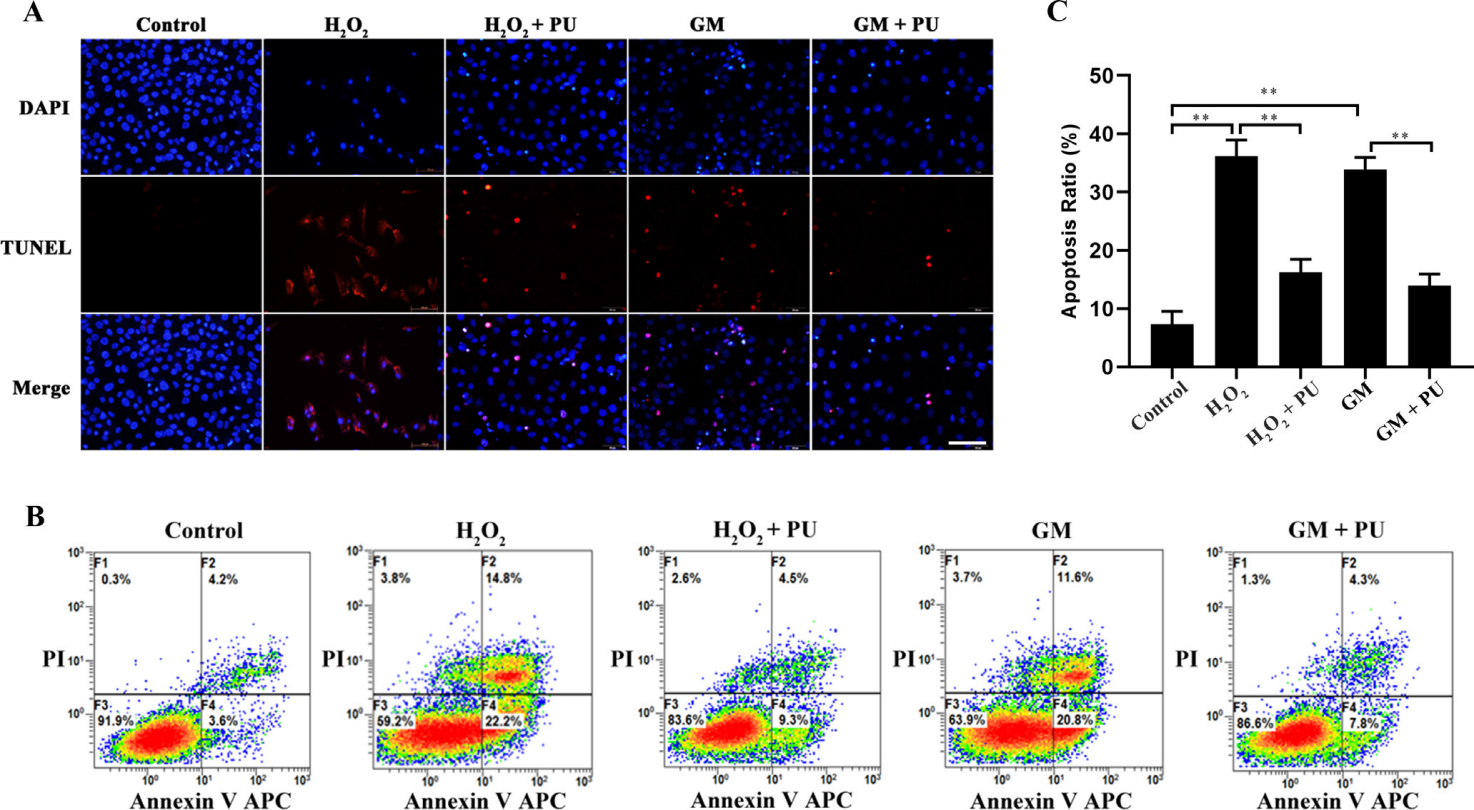
Rate of apoptosis after different treatments as determined by TUNEL assay and flow cytometry. (A) Image of HEI-OC1 cells stained with TUNEL staining. Nuclei were stained with DAPI (blue) and HEI-OC1 cells were stained with TUNEL (red). Scale bar, 100 µm. (B) Flow cytometry was used to assess apoptosis after treatment under different conditions for 24 h. (C) The apoptotic rate of the cells was statistically analysed. n=6. **P<0.01. PU, puerarin; GM, gentamicin; HEI-OC1, House Ear Institute-Organ of Corti 1.

